# Genetic Variation versus Morphological Variability in European Peatland Violets (*Viola epipsila*—*V. palustris* Group)

**DOI:** 10.3390/biology12030362

**Published:** 2023-02-24

**Authors:** Justyna Żabicka, Tom Kirschey, Grzegorz Migdałek, Aneta Słomka, Elżbieta Kuta

**Affiliations:** 1 Department of Plant Cytology and Embryology, Institute of Botany, Faculty of Biology, Jagiellonian University in Kraków, 9 Gronostajowa St., 30-387 Cracow, Poland; 2 International Peatland and Southeast Asia Programme, International Department, The Nature and Biodiversity Conservation Union (NABU), 3 Charitéstrasse, 10117 Berlin, Germany; 3 Institute of Biology and Earth Sciences, Pedagogical University of Cracow, 2 Podchorążych St., 30-084 Cracow, Poland

**Keywords:** genetic diversity, hybridization, species delimitation, gene sequences, RAD-Seq, molecular markers, genome size

## Abstract

**Simple Summary:**

The wetland violets of Central and Northern Europe (*Viola epipsila* Ledeb., *V. palustris* L.) are endangered because the ranges of both species are drastically decreased due to global climatic changes. Their disappearance might also be the result of the formation of interspecific hybrids which can replace the parent species. The study of such species is particularly important because they might be considered as indicators of anthropogenic changes occurring in peatlands and their disappearances. The taxonomic situation of studied species is intricate, and the presence of interspecific hybrids and putative introgressants [*V. pubifolia* (Kuta) G. H. Loos (=*V. palustris* subsp. *pubifolia* Kuta)] makes it even more complicated. The main goal of our study was to reconstruct the origin of *V. pubifolia* and its genetic relatedness to both putative parental species—*V. palustris* and *V. epipsila*—using advanced molecular methods. The taxonomic problem has been finally solved. We found no basis to separate *V. pubifolia* as a species in its own right because its morphological characters as well as genetic variation fall well within the range of variability of *V. palustris*. We have also concluded that the low genetic differentiation and heterozygosity of *V. epipsila* in Europe might be a cause of the reduced tolerance of this species to changing environmental conditions and can possibly lead to its extinction.

**Abstract:**

In Europe, the *V. epipsila*—*V. palustris* group comprises *V. epipsila* Ledeb., *V. palustris* L., *V. pubifolia* (Kuta) G. H. Loos (=*V. palustris* subsp. *pubifolia* Kuta), interspecific hybrids, and putative introgressants. The genetic affinity of *V. pubifolia* to *V. palustris*, and their shared origin via hybridization followed by polyploidization, were confirmed using inter simple sequence repeat (ISSR) markers, restriction site-associated DNA sequencing (RAD-Seq), and a low-copy nuclear gene, *GPI*, which encodes glucose-6-phosphate isomerase. The other taxa of subsect. *Stolonosae* were not identified as putative parents of *V. pubifolia* by *GPI*. Our analyses indicated that *V. pubifolia* can be included in the morphological and genetic variation of *V. palustris*. The ISSR, RAD-Seq, and genome size value separated well *V. palustris* from *V. epipsila* and hybrids. The results also reopen the discussion on intraspecific variation in the context of taxa ranks and species concepts. The reduced tolerance of *V. epipsila* in Europe to changing environmental conditions might result from low genetic differentiation and heterozygosity, as well as the increased number of interspecific hybrids (*V. epipsila* × *V. palustris*), and eventually can possibly lead to its extinction. The disappearance of populations/individuals of this species may indicate anthropogenic changes occurring in peatlands.

## 1. Introduction

*Viola* L. is one of the largest angiosperm genera, comprising ca. 658 recognized species; it consists of two sub-genera, 31 sections, and 20 subsections. It is distributed mainly in the temperate zones of both hemispheres and it occupies a high diversity of habitats and elevations [1]. The taxonomy of the genus is complicated due to interspecific hybridization commonly occurring between closely related species of the same section, especially in disturbed or transitional habitats, and polyploidization, which plays a key role in its evolution. Both processes have enhanced the population genetic variation, leading to intraspecific variability and speciation [1,2,3,4,5,6,7,8,9,10,11,12,13,14,15,16,17,18,19,20,21].

European peatland violets form a taxonomically intricate group comprising the tetraploid *V. epipsila* Ledeb. (2*n* = 4*x* = 24), the octoploids *V. palustris* L. and *V. pubifolia* (Kuta) G. H. Loos (=*V. palustris* subsp. *pubifolia* Kuta; 2*n* = 8*x* = 48), the interspecific hexaploid (2*n* = 6*x* = 36) F_1_ hybrids (*V. ruprechtiana* Borb., syn. *V. fennica* F. Nyl.), and putative introgressive forms ([1] and references therein, [8,9,10,17,22]). These taxa belong to the subsect. *Stolonosae* Kupffer of the sect. *Plagiostigma* Godr., the oldest (crown node of 16.6 Ma) and the most species-rich (139) of all allopolyploid *Viola* sections [1]. The subsect. *Stolonosae* (crown node age c. 12.7 Ma) with 39 allopolyploid species (4*x*, 8*x*) has mainly a north-temperate distribution. The delimitation of this subsection is based on the occurrence of allopolyploids between distantly related internal lineages, one of which supposed to be *V. palustris*, the type of the subsection. Several allo-octoploids with boreal distributions are young taxa dated on 2.5–5 Ma, suggesting their origin is in response to the climate cooling and repeated glaciations in the Pleistocene [1].

*Viola epipsila* is a circumboreal species ranging from western Siberia to northwestern North America with scattered locations in Northern European countries (Norway, Finland, Iceland) [1,22,23,24,25,26,27,28]. *Viola palustris* is an amphi-Atlantic species and is distributed as far east as the Ural Mountains. The intraspecific ranks of species and new species delimitations in the subsect. *Stolonosae* are disputable and discussed in the revised phylogenetic classification of the genus *Viola* [1].

**Figure 1:**
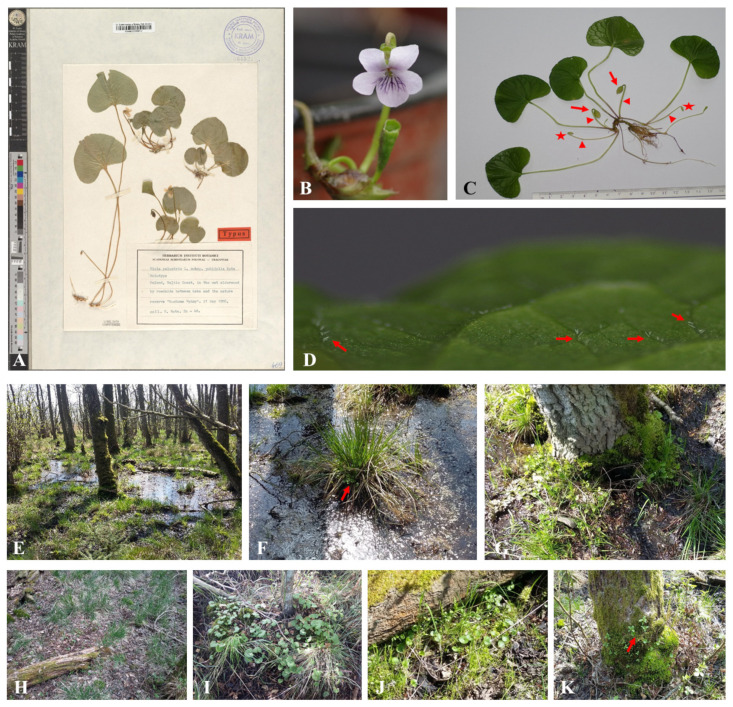
Morphological features of *V. pubifolia* and occupied habitats. Herbarium specimens of *V. palustris* L. subsp. *pubifolia* Kuta described for the first time from Słowiński National Park [10] (**A**). Face view of CH flower (**B**); capsule of CH flower (arrows), bud-like, fully developed CL flower (asterisks), visible bracts on the pedicel (arrowheads) (**C**); lower leaf surface with visible hairs marked with arrows (**D**). Occupied habitats: wet, periodically flooded areas (**E**–**G**) or less humid places (**H**–**K**): in clumps of grass (**F**), close to trees (**G**), close to rotten trunks (**J**), on growing tree trunks partly covered with moss (**K**). CH—chasmogamous flower; CL—cleistogamous flower; arrows—individuals on particular locality.

*Viola epipsila* and *V. palustris* both occur in wetlands, although *V. epipsila* prefers rather eutrophic groundwater-influenced fen habitats and is associated with wet alder stands; *V. palustris*, with a wider ecological tolerance, occurs in peatland vegetation, in wet meadows, and in pastures [10,17,29,30]. The overlapping ranges and flowering of chasmogamous (CH) flowers of these closely related allopolyploids, which share a genome, weakens the isolation barriers, creating suitable conditions for interspecific hybridization, and leading to the genetic diversity of the complex [1,9,10,11,30]. The wetland areas have drastically decreased because of the combined effect of climate change and drainage. As a consequence, the habitats of both species are disappearing and their ranges, especially that of *V. epipsila*, are declining in Central Europe [30]. These species may be considered as indicators of the state of the peatlands they occupy; the decreasing numbers of individuals or the disappearance of whole subpopulations of both species indicate a large-scale change in the wetland and peatland habitats. In sympatric European populations of both species, previous studies have identified interspecific hexaploid (2*n* = 6*x* = 36) F_1_ hybrids, putative introgressive forms, and the hybrid species *V. pubifolia* (Kuta) G.H. Loos [8,9,10,11,30,31]. Vigorous interspecific hybrids may be replacing pure individuals of *V. epipsila* in European populations because they may have adaptations which help them survive in a changing environment [1,9,10,30,31,32,33,34]. Hybrids with intermediate characters between parental species may be erroneously described as *V. epipsila*, as was confirmed by a critical revision of Polish and European herbarium specimens [10]. A similar difficulty concerns the designation of *V. pubifolia* individuals differing in some morphological features from *V. palustris* (e.g., scattered hairs on the lower leaf surface, location of bracts in the middle of the pedicel, leaf shape, flower size), described for the first time in Słowiński National Park [10] (Figure 1A–D). Based on the morphological and cytological (2*n* = 8*x* = 48) features and the reproduction system (fully fertile) [8,9], these forms were distinguished as a subspecies of *V. palustris* (*V. palustris* L. subsp. *pubifolia* Kuta) [10] and later named as *V. pubifolia* (Kuta) G. H. Loos [31]. Plants with some features of *V. pubifolia* have been identified from other localities in Poland and Germany. In some populations, they occur sympatrically with typical *V. palustris* [10]. Currently, *V. pubifolia* is easy to find in Słowiński National Park, where it grows in very wet, periodically flooded areas (Figure 1E–G), which are often associated with clumps of sedges and grasses (Figure 1F) or close to trees (Figure 1G), but also in less humid places (Figure 1H–K), close to rotten tree trunks (Figure 1J), and even growing on tree trunks partly covered in moss (Figure 1K). In Słowiński National Park, *V. pubifolia* is found in several vegetation assemblages that have been classified according to the GEST approach, linked to water table and greenhouse gas emissions before and after peatland restoration measures [35].

The origin and genetic relatedness of *V. pubifolia* to both putative parental species has never been confirmed with the use of molecular markers. As the taxonomic position of this taxon is still questionable, the present research focused on (1) evaluating the genetic diversity of *V. pubifolia* and its genetic affinity to *V. palustris* from Central and Northern Europe, using inter simple sequence repeat (ISSR) markers and next-generation sequencing technology (restriction site-associated DNA sequencing, RAD-Seq); (2) reconstructing the origin of *V. pubifolia* with the use of a low-copy nuclear gene, *GPI*, which encodes glucose-6-phosphate isomerase; and (3) establishing the relationship of *V. pubifolia* with closely related violets of subsect. *Stolonosae.*

## 2. Materials and Methods

### 2.1. Plant Material Collection

**Figure 2:**
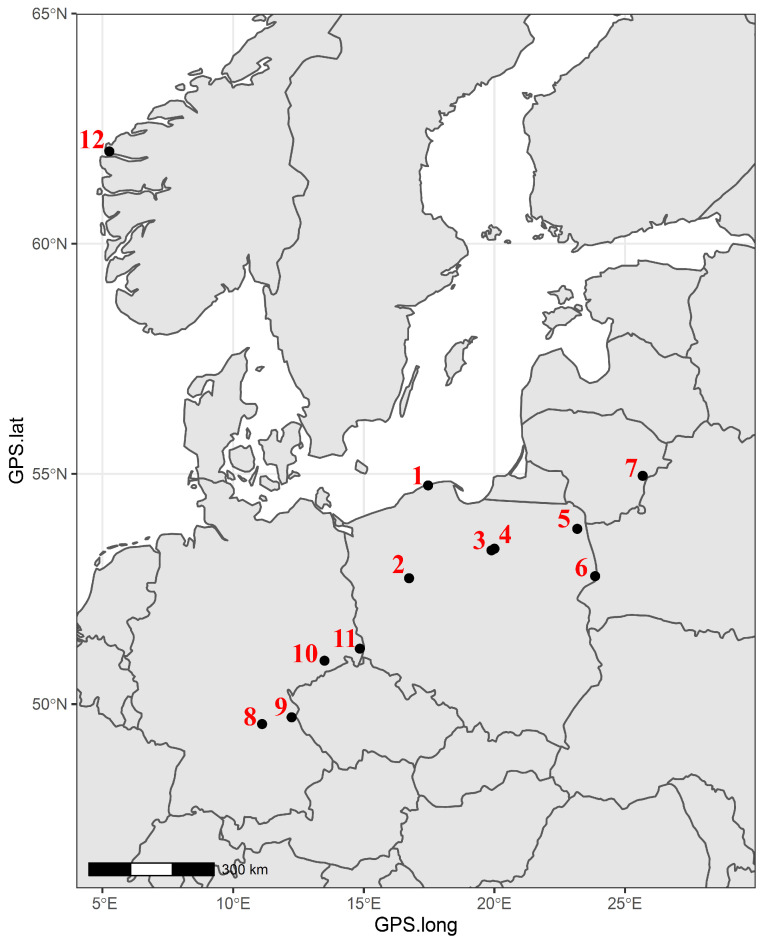
Geographical distribution of Central and Northern European studied populations of *V. pubifolia*, *V. palustris*, *V. epipsila*, and hybrids (*V. epipsila* × *V. palustris*): 1—Słowiński National Park (NW Poland), 2—Połajewo near Poznań (W Poland), 3—Ostrów Tarczyński Nature Reserve (NE Poland), 4—Szczupliny (NE Poland), 5—Balinka (NE Poland), 6—Białowieża National Park (NE Poland), 7—Pravalas Botanical Reserve (E Lithuania), 8—“Dormitzer Forst” Kalchreuth (SE Germany), 9—Floß (SE Germany), 10—Grillenburg (E Germany), 11—Oberlausitz (E Germany), 12—Barmøya (SW Norway). Detailed information of sample designation is in Table S1.

The plant material originated from 12 sites from 4 countries (Germany, Lithuania, Norway, Poland) in Central and Northern Europe (Table S1, Figure 2). For all sampling, legal permits by the authorities had been obtained. *Viola pubifolia* plants were randomly collected in the “Mierzeja” nature reserve (a dune forest strict reserve) in the Słowiński National Park (SNP), Poland. Plants of *V. palustris*, *V. epipsila*, interspecific hybrids (*V. epipsila* × *V. palustris*), and samples defined as an introgressive form towards *V. palustris* were also included in the molecular analysis. The sample identification was based on different criteria: pre-selected morphological features but also ISSR markers, pollen viability, genome size value, especially important in F_1_ hybrid, and introgressant identification (Table S1) [10,30]. Randomly selected plants of *V. pubifolia* and *V. palustris* were gathered in natural populations and transferred to the experimental garden condition (located in Cracow, Ugorek district; 50.078320; 19.986784) for observation and further investigation.

For molecular analysis, two to three healthy and fully developed leaves per plant of *V. pubifolia* and *V. palustris* were harvested randomly from plants growing in natural sites. Thirty samples of *V. pubifolia* and one sample of *V. palustris* were taken from plants located in the experimental garden (Table S1, Figure 2). The distance of a minimum ca. 4 m between the individuals growing in nature was kept to avoid clonality. The leaves were stored in sterile tubes (F.L. MEDICAL, Torreglia, Italy) filled with silica gel (F.H.U. “DOR-CHEM”, Kraków, Poland) at room temperature not exceeding 25°C. The DNA of six *V. palustris* (BAL_7, BAL_9, BAL_10, L_18, L_19, L_20), three *V. epipsila* (SZ_4, SZ_6, SZ_7), three hybrids (OST_1, OST_3, OST_5), and three *V. pubifolia* (N_B!2, N_B!3, N_B!5) samples were used from the previous research [30] (Table S1). Plants growing in nature reserves, national parks, and law-protected *V. epipsila* were collected with permission in accordance with the relevant institutions. The voucher specimens were deposited in the Herbarium of the Institute of Botany of the Jagiellonian University in Kraków, Poland (KRA, accession numbers: 0552067-0552072, 602281-602286).

### 2.2. Genome Size Assessment

In total, 2 to 3 fresh leaves per plant of 39 *V. pubifolia* samples were harvested and their nuclear DNA content was estimated using flow cytometry (FC). Genome size analyses were performed based on previously the described protocols ([30] and references therein). The genome size values of *V. palustris*, **V. epipsila*,* and hybrids (*V. epipsila* × *V. palustris*) added to Table S2 were taken from Żabicka et al. [30].

### 2.3. DNA Extraction

DNA extractions from silica dried leaves, as well as quality assessments, were conducted following the protocol of Żabicka et al. [30]. In total, 121 samples of DNA were isolated and different number of samples were used for ISSR (83 samples), *GPI* analysis (one sample), RAD-Seq (21 samples), and genome size assessment (39 samples).

### 2.4. Morphological Features of V. epipsila—V. palustris Group

Based on different floras, published papers, and authors’ observations, the morphological characteristics of *V. epipsila*, *V. palustris*, interspecific hybrids (**V. epipsila* × V. palutris*), and *V. pubifolia* were compiled (Table S3).

### 2.5. ISSR Analysis

Ten primers of ISSR markers were chosen based on amplification effectiveness and the number of polymorphic products [36,37] (Table S4). For the ISSR analysis, 83 samples were included in total: 62 samples of *V. pubifolia*; 15 samples of *V. palustris*; 3 samples of *V. epipsila*; 3 samples of hybrids (*V. epipsila* × *V. palustris*). To balance the sample size, two types of analysis were performed: (A) *V. pubifolia* from SNP (the number of samples was reduced to 20), *V. palustris* from different populations (Balinka, Poland; Floß, Grillenburg, Oberlausitz, Germany; Pravalas Botanical Reserve, Lithuania; Barmøya, Norway), *V. epipsila* (Szczupliny, Poland), and hybrids *V. epipsila* × *V. palustris* (Ostrów Tarczyński Nature Reserve, Poland); (B) 62 samples of *V. pubifolia* from SNP. PCRs and electrophoresis on agarose gel, band patterns observation, and capturing were conducted using chemicals, protocols, laboratory equipment, and software described in Żabicka et al. [30]. The annealing temperature was tested and depended on the primer (Table S4).

POPGENE v. 1.32 [38] and FAMD v. 1.31 [39] were used to analyze ISSR polymorphism and genetic diversity. A split phylogenetic network (NeighborNet) in SplitsTree v. 4.6 [40] based on the Dice coefficient was constructed to evaluate the relationships between *V. pubifolia* and other studied individuals, species, and populations (A), and also to examine the diversity of *V. pubifolia* populations (B). A bootstrap was calculated on 2000 replicates. The STRUCTURE analysis [41] assumed an admixture between populations, and correlated allele frequencies between the clusters. In total, 5 independent runs were performed for each K value (1–5) with burn-in of 10^5^ and 10^6^ Markov chain Monte Carlo replicates after burn-in. The optimal K value was chosen based on the mean logarithmic likelihood of K values and ΔK values [42], calculated using Structure Harvester v. 0.6.94 [43]. The clustering results were summed up in CLUMPAK [44] with LargeKGreedy search method and 2000 random input order repeats. To evaluate statistical significance of clustering, a hierarchical analysis of molecular variance (AMOVA) was performed on groups suggested by STRUCTURE using Arlequin v. 3.5.2.2 [45].

### 2.6. A Low-Copy Nuclear Gene GPI Analysis

To discover the phylogeny of *V. pubifolia* and its relations with *V. palustris* and other species from *Plagiostogma* section, highly conserved *GPI* gene was sequenced according to the previous studies on reconstructing reticulate phylogeny of allopolyploid violets [18] and to discriminate a new species [46].

The sequences of two homoeologs of *GPI* (CHAM—*GPI-C*, MELVIO—*GPI-M*) of *V. pubifolia* were obtained in two steps: (1) PCR amplification reactions with pairs of primers; sequencing of PCR product with the use of primers covering exon12–exon16 or exon13–exon18 of the GPI locus. The PCR and sequencing primers, composition of reaction mixture, and PCR program were taken from Marcussen et al. [18]. (2) Based on the sequences obtained in step one, we designed sixteen primers specific for two homoeologs according to procedure by Scheen et al. [47] (Table 1) covering exon12–exon16 and exon13–exon17 GPI locus. The amplifications were conducted according to the touchdown PCR program: initial denaturation at 94 °C for 5 min, 26 cycles: denaturation 94 °C, 30 s; annealing temperature starting from 62.5 °C (gradually decreased by 0.5 °C per cycle), 30 s; elongation at 72 °C, 60 s, and then 21 cycles: denaturation at 94 °C, 30 s; annealing temperature at 49 °C, 30 s; elongation at 72 °C, 60 s; final extending at 72 °C, 10 min and subsequent hold at 6 °C. The amplifications in steps 1 and 2 were conducted in Bio-Rad T100 Thermal Cycler (Bio-Rad, Hercules, CA, USA) using Phusion HF buffer, Phusion polymerase, and dNTPs obtained from Thermo Scientific, Waltham, MA, USA. The PCR products were separated in 1% agarose gel with 1× TBE and SimplySafe (EURx Sp. z o.o., Gdańsk, Poland) for about 90 min at 120 V. Bands were observed and captured with a MultiDoc-It imaging system with VisionWorks^®^ LS Analysis Software (UVP, Upland, CA, USA).

For the sequencing of a *GPI,* we have chosen only the samples which gave the best quality PCR products (Table S1). The sequencing was performed by Genomed SA, Warsaw, Poland. The sequences were manually checked for quality using BioEdit v. 7.2 [48], assembled into contigs using CAP [49], and aligned in MUSCLE [50]. The sequences of two homoeologs of *GPI* (*GPI-C*, *GPI-M*) of *V. palustris*, *V. epipsila*, *V. pluviae*, *V. blanda*, *V. primulifolia*, *V. jalapaensis*, *V. lanceolata*, *V. minuscula* [=*V. pallens* auct., non (Banks) Brainerd)], *V. occidentalis*, *V. macloskeyi*, *V. renifolia*, *V. principis*, *V. vaginata*, *V. selkirkii*, *V. mirabilis*, *V. rubella*, *V. pusilla*, and *V. congesta* were taken from GenBank accessions [18].

Phylogenetic inference was performed in MrBayes v. 3.2.6 [51]. Data were partitioned into GPI nucleotide sequences and indels and coded with a simple coding method [52] in SeqState v. 1.4.1 [53]. The HKY model was chosen based on the results of BIC calculation in jMODELTEST v. 2.1.10 [54]. The indels were treated as restriction data with variable coding. In total, 2 runs were performed (4 chains total) with 3 million MCMC generations, to ensure the analysis reached convergence, and 25% trees were discarded as burn-in. The final trees were visualized in FigTree v. 1.4.3 [55].

### 2.7. RAD Sequencing and Bioinformatics Analysis

In total, 21 selected samples of DNA (Table S1) were normalized to 10 ng/μL and submitted to Floragenex, Inc. (Eugene, OR, USA). RAD-Seq was performed according to the protocol by Baird et al. [56], with a single digestion of total DNA with SbfI restriction endonuclease enzyme. After digestion, the samples were heat-inactivated for 20 min at 80°C, followed by the addition of modified Solexa^©^ adapters (Illumina, Inc., San Diego, CA, USA). The samples with ligated adapters were purified and amplified with 50 μL 2× Phusion Master Mix (NEB, Ipswich, MA, USA), 5 μL of 10 μM modified Solexa^©^ Amplification primer mix (Illumina, Inc., San Diego, CA, USA) and up to 45 μL H_2_O. The library was quantified with a Qubit fluorometer and run on an Agilent Bioanalyzer with the High Sensitivity kit to determine the size distribution, which was 505 bp. Furthermore, 1 × 150 bp single-end sequencing was performed on the HighSeq 3000 at the Oregon State University Center for Quantitative Life Sciences.

Genotyping and SNP calling was performed in STACKS v. 1.44 [57]. Reads pre-processing and quality filtering was performed in the process_radtags module in STACKS with the default parameters. Loci assembly was conducted using denovo_map pipeline with the minimum number of matching raw reads set to 10 [58]. The maximum number of nucleotide mismatches within and between individuals was determined by testing for asymptotic distribution [59] and was set to 6. The loci present in at least 70% of the individuals were extracted using the populations program along with parametric data. An individual-level pairwise genetic distance matrix was generated and visualized in R v. 4.0.5, based on Peakall-Smouse coefficient, calculated using PopGenReport [60]. Additionally, we calculated the SNP-based pairwise *F_st_* at the species level in the populations program. To evaluate the degree of shared ancestry between *V. pubifolia*, hybrids, and parental species, we performed an analysis based on a model of the nearest neighbor coancestry implemented in fineRADstructure [61]. The output data from Stacks de novo pipeline was reformatted for RADpainter using the Stacks2fineRAD.py script [61], allowing for a maximum of 10 SNPs per locus and 30% of the missing loci to be included in the analysis. To generate a coancestry matrix in RADpainter, we assumed an 8N ploidy for the studied species. A fineSTRUCTURE MCMC analysis was run with 100,000 burn-in and 1,000,000 sample iterations keeping every 1000th sample. The tree was built from the minimum state with burn-in set to 10,000. We developed a custom R script for the counting of common haplotypes between selected samples, based on the populations program output. Consensus haplotypes were filtered out and the remaining haplotypes intersected between *V. epipsila*, *V. palustris*, **V. pubifolia*,* and hybrid samples.

## 3. Results

### 3.1. Genome Size and Genetic Differentiation of V. pubifolia vs. V. palustris

The genome size estimate for *V. pubifolia* was equal to 4.14 pg and thus was similar to the estimate obtained for *V. palustris* (4.26 pg). *Viola epipsila* had a smaller genome (2.52 pg) than *V. palustris*, confirming octoploid and tetraploid ploidy of the two closely related species, respectively. The genome size (3.42 pg) of the interspecific hybrid (*V. epipsila* × *V. palustris*) was intermediate between the parental species but differed significantly from the genome of *V. pubifolia* (Table S2).

The ISSR analysis of the selected 20 *V. pubifolia* specimens from SPN, 15 plants of *V. palustris* from different regions, 3 samples of **V. epipsila* × *V. palustris** hybrids, and 3 samples of *V. epipsila* resulted in 201 resolved bands. The analysis of 62 specimens from SPN resulted in 143 resolved bands. The number of polymorphic bands was higher in the group of all *V. palustris* (54 bands—26.87%) than in the remaining samples of *V. pubifolia*. Nei’s gene diversity (*H_j_*) was higher for *V. palustris* (0.09) than for *V. pubifolia* (0.05). The total gene diversity (*H_T_*) reached 0.09 and the mean gene diversity (*H_S_*) within populations was slightly lower (0.07). The highest gene diversity (*G_ST_*) was between the populations (0.21) (Table 2A). The group containing only *V. pubifolia* samples had 30.85% polymorphic bands and Nei’s gene diversity of *H_j_* = 0.06 (Table 2B).

**Figure 3:**
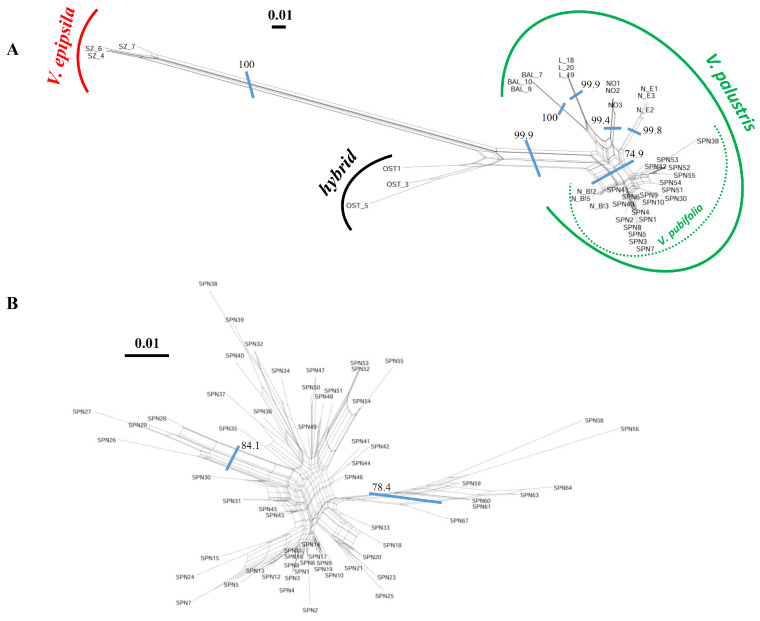
NeighborNet analysis of: (**A**)—*V. pubifolia* (SPN, N_B!), *V. palustris* (BAL, L, NO, N_E), *V. epipsila* (SZ), *V. epipsila* × *V. palustris* hybrid (OST); (**B**)—*V. pubifolia* (62 samples) based on Dice coefficient from ISSR data. Bootstrap analysis was performed on 2000 replicates. Origin of samples: Germany (N), Poland (SNP, BAL, OST, and SZ), Lithuania (L), and Norway (NO). Detailed information in Table S1.

Based on NeighborNet analysis performed on the Dice coefficient genetic distance matrix, the *V. epipsila*, *V. palustris*, **V. pubifolia*,* and hybrid (**V. epipsila* × *V. palustris**) samples formed three groups. The first group corresponded with *V. palustris* and *V. pubifolia*, the second with *V. epipsila*, and the third with the hybrid (**V. epipsila* × *V. palustris**). The split separating *V. epipsila* (BS 100) and both *V. palustris* and *V. pubifolia* (BS 99) showed high bootstrap values (BSs). The samples from different populations within groups were also separated by strongly supported splits (Figure 3A). NeighborNet analysis of 62 *V. pubifolia* samples resulted in weak differentiation within the group; only two small groups of the samples were separated and the distance between them and other samples was very low (Figure 3B).

**Figure 4:**
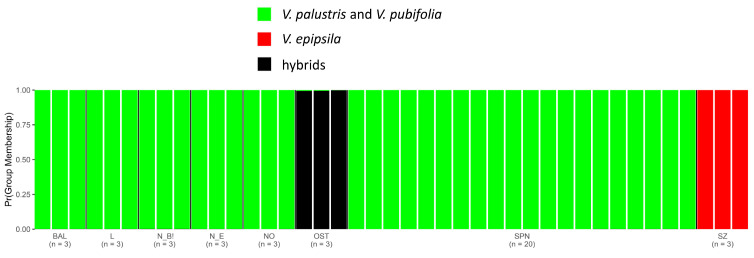
Results of ISSR analyses of 8 populations of *V. pubifolia*, *V. epipsila*, *V. palustris*, and *V. epipsila* × *V. palustris* hybrid STRUCTURE at K = 3. Origin of samples: Germany (N), Poland (SNP, BAL, OST, and SZ), Lithuania (L), and Norway (NO). Detailed information in Table S1.

STRUCTURE Bayesian analysis assumed there were three groups in all studied taxa, K = 3 (ΔK = 79.75; Figure S1). The first group contained all samples of *V. pubifolia* (SPN, N_B!) and *V. palustris* (N_E, NO, L, BAL). *Viola epipsila* (SZ) and **V. epipsila* × *V. palustris** hybrid (OST) formed two separate groups (Figure 4).

### 3.2. RAD-Seq Analysis Confirmed the Genetic Similarity of V. pubifolia and V. palustris

RAD sequencing yielded a total of 9.36 × 10^6^ reads per sample (SD 4.43 × 10^6^). The average sequencing coverage per individual was 1800.3×. After STACKS denovo analysis and filtering, 1109 loci were retained, including 689 SNPs. Missing data were present in 13 samples and its proportion did not exceed 30%. Heterozygosity (*H_o_*) of hybrid was the highest (0.72), of European *V. epipsila* was the lowest (0.02), and *V. pubifolia* (0.48) and *V. palustris* (0.46) represented relatively high heterozygosity levels. The mean inbreeding coefficient of an individual relative to the subpopulation was positive only for *V. epipsila* (0.02; Table 3). The highest genetic distance (*F_st_*) was between *V. epipsila* and *V. palustris* or *V. pubifolia*; the lowest was between *V. palustris* and *V. pubifolia* (Table 4).

**Figure 5:**
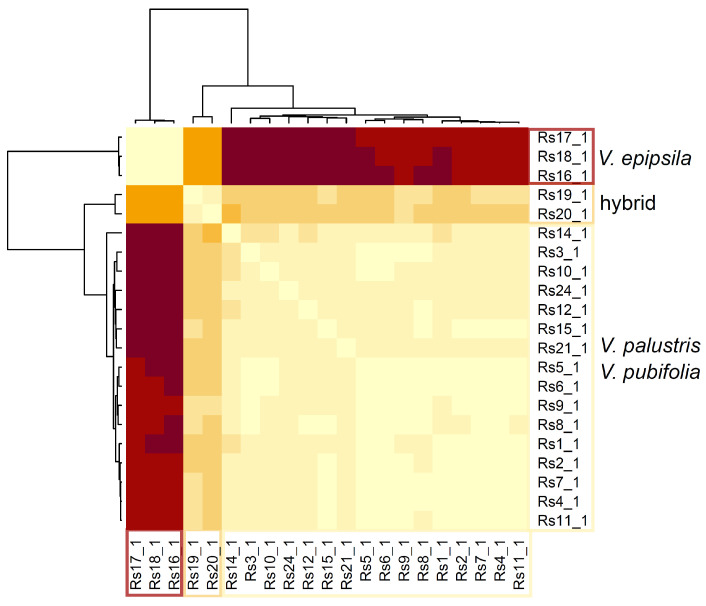
Heatmap based on RAD-Seq pairwise Peakall-Smouse genetic distance between *V. epipsila*, *V. palustris*, *V. pubifolia*, and interspecific hybrid (*V. epipsila* × *V. palustris*). Intensity of colors indicates the distance between taxa—the more intense the color, the higher the distance between taxa. Detailed information of sample designation in Table S1.

**Figure 6:**
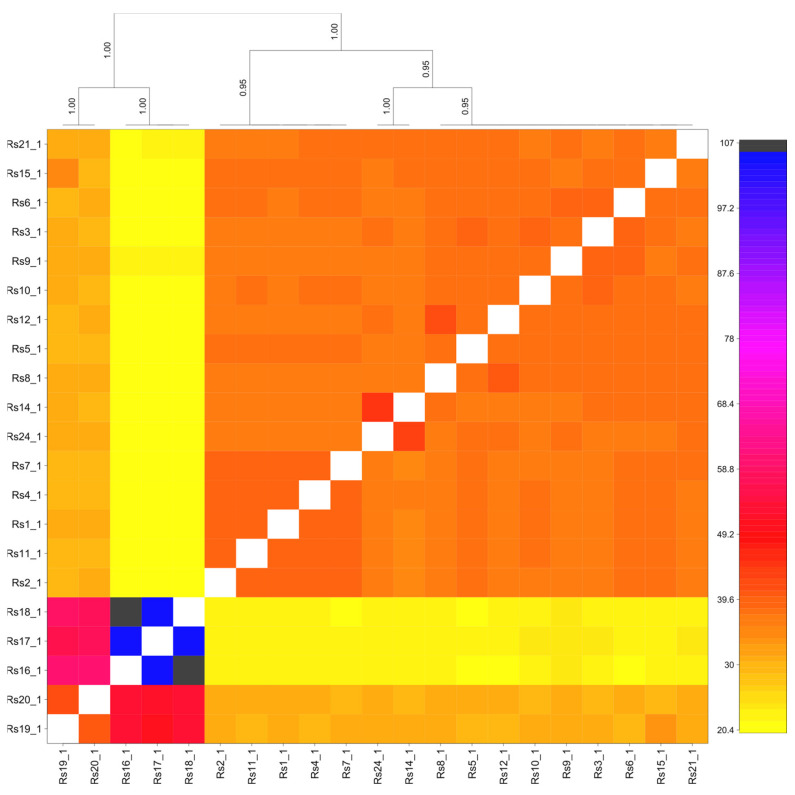
Heatmap based on a clustered coancestry matrix, generated during fineRADstructure analysis and showing clustering of individuals belonging to *V. epipsila*, *V. palustris*, and their hybrids. Darker colors denote increasing levels of coancestry.

The heatmap based on the pairwise Peakall-Smouse genetic distance clearly indicated low genetic distance between *V. pubifolia* and *V. palustris* (Figure 5). FineRADstructure results revealed two distinct groups, corresponding to *V. epipsila* and *V. palustris*, with average coancestry within groups of 105.0 and 39.7, respectively. Hybrid samples were more similar to the *V. epipsila* group (coancestry coefficient = 58.5) than *V. palustris* (coancestry coefficient = 30.3) (Figure 6).

### 3.3. Common Haplotypes of Studied Taxa

RAD-Seq analysis showed the mean number of shared haplotypes between the studied taxa: 93.00 for *V. epipsila* and hybrids; 48.00 for *V. palustris* and *V. epipsila*; 87.83 for *V. pubifolia* and *V. epipsila*.

More detailed analysis of the common haplotype number of *V. pubifolia* with other taxa, taking into account the origin of individual samples, indicated that the mean number of common haplotypes of *V. pubifolia* and *V. epipsila* was similar for Polish and Lithuanian specimens (113.54, 116.08 and 118.46). The differences in the mean common haplotype numbers between *V. pubifolia* and *V. palustris* were related to the origin of the material—514.46 with samples from Germany and 577.54 from Lithuania (Table 5).

### 3.4. GPI Homoeologs of V. pubifolia

**Figure 7:**
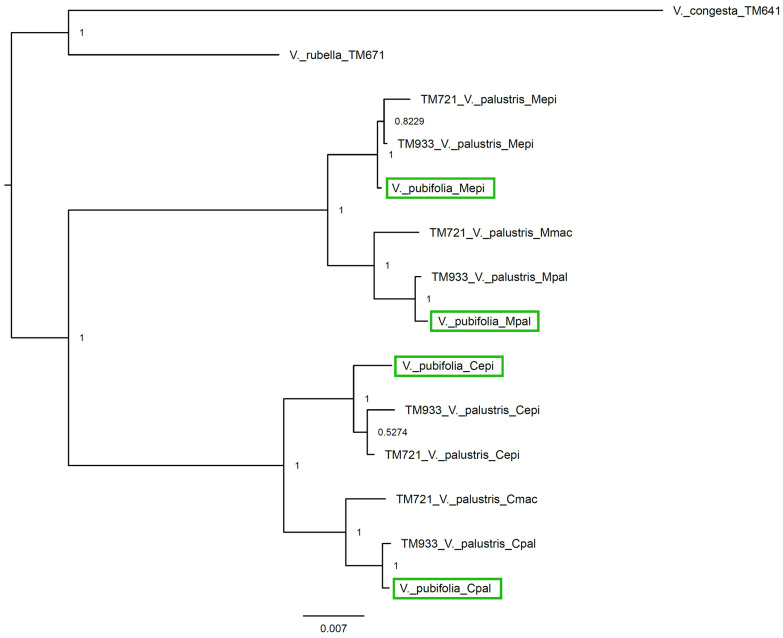
Phylogenetic tree of *V. palustris* and *V. pubifolia* based on sequences of two homoeologs of *GPI* (*GPI-C*, *GPI-M*) and constructed using Bayesian method. Node labels present clade support probability.

**Figure 8:**
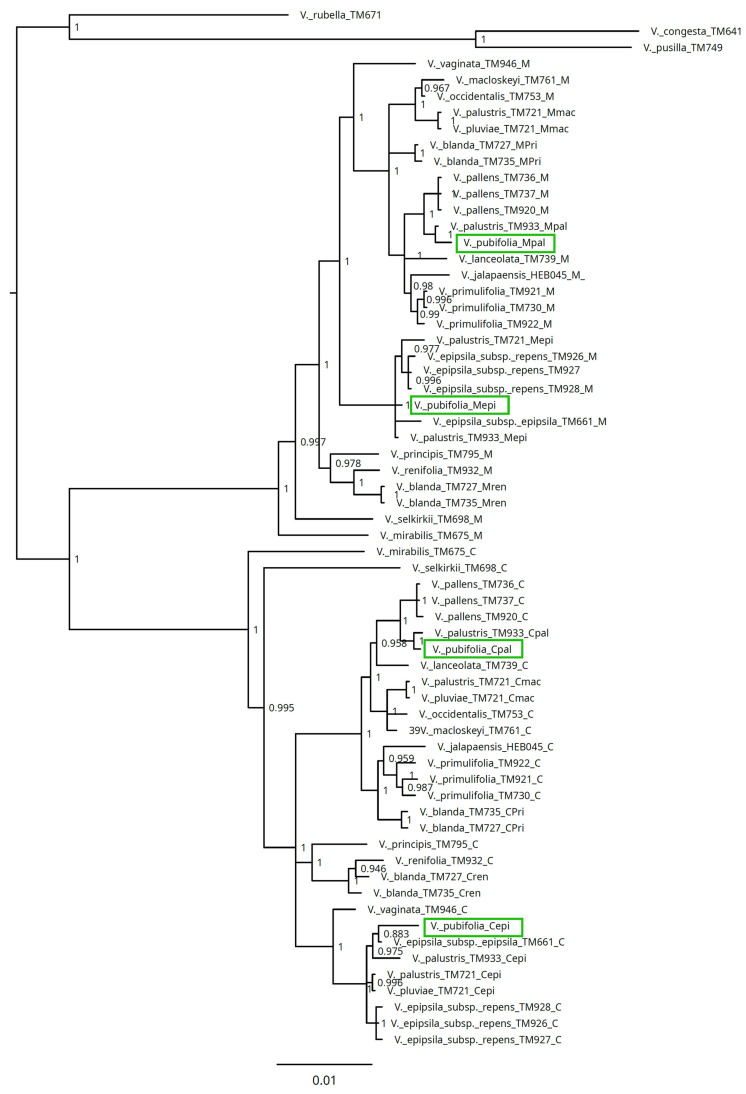
Phylogenetic tree of sect. *Plagiostigma* subsect. *Stolonosae* including *V. pubifolia* and the outgroups (*V. selkirkii* of subsect. *Patellares*; *V. mirabilis* of sect. *Viola*; *V. rubella* of sect. *Rubellium*; *V. pusilla* sect. *Andinium*; *V. congesta* of sect. *Andinium*) based on sequences of two homoeologs of *GPI* (*GPI-C*, *GPI-M*) and constructed using Bayesian method. Node labels present clade support probability. *V. pallens* = *V. minuscula*, according to Marcussen et al. [1].

The analysis of highly conserved *GPI* showed that *V. pubifolia* has two of the same homoeologs as *V. palustris*: (Figure 7). The first homoeolog is shared with *V. epipsila* subsp. *epipsila* (Cepi, Mepi), and second with *V. minuscula* (=*V. pallens*; Cpal, Mpal; Figure 8).

### 3.5. Phenotypic Plasticity of V. palustris vs. V. pubifolia Variability

Based on the literature and on the authors’ observation, the intraspecific morphological variability of *V. palustris* has been expressed by describing the subspecies, varietas, and forms [11,17,18,23,24,25,26,27,46,63]. The characteristics of *V. palustris* from the data in the Table S3 show the range of variability of qualitative and quantitative features of vegetative and generative organs. For leaves, the features include length/width ratio: 0.54–1.17; predominate forms with glabrous lower surface, but also specimens with hairy leaf lower surface (varying in hair number); shape of apex mostly obtuse, but also leaves with an acute or subacute apex; type of margin: crenate; petiole glabrous or sometimes slightly hairy. For flowers, the features include length of CH sepals with calycine appendages: 3.5–7.0 mm; length of CH spur: 0.5–1.5 mm; length of CH lower petal with spur: 8–14 mm; mostly hairy lateral petals but flowers with glabrous lateral petals also found; ratio of distance of bracts from rhizome to length of pedicel: 0.20–0.60, bracts located mostly in the lower half of pedicel but sometimes also in the middle of the pedicel or slightly above; pedicels of CH flowers glabrous or sometimes with scattered hairs; chromosome number/ploidy: 48/8*x*. Fruits (capsules) are filled with seeds (fully fertile), pollen is viable (Table S3).

The morphological characteristics of *V. pubifolia* can be included in *V. palustris* variability (Table S3).

## 4. Discussion

Based on ISSR, *GPI*, RAD-Seq, and genome size values, *V. pubifolia* can be included in the *V. palustris* genetic variation. The applied molecular techniques resolved the origin of *V. pubifolia* and reopened the questions of the taxonomical ranks of intraspecific variation and concept of species—are they morphological, molecular, or integrative? In this context, the rank of studied taxon *V. pubifolia* has to be revised. Neither the morphological nor the genetic intraspecific variation of *V. palustris* entitles the distinguishing of this variability in the rank of a subspecies (*V. palustris* subsp. *pubifolia*) or a species (*V. pubifolia*). These results are in line with the recent revision of the genus *Viola*, in which intraspecific taxa (subspecies, varietas) of *V. epipsila*—*V. palustris* group were considered as synonyms of these two species and were included into their variability or were delimitated as new species. Referring to the study of the *V. epipsila*—*V. palustris* group, phylogenetic studies indicate that a relatively narrow species concept coinciding with morphological geographic units best apply to the taxa of the *Stolonosae* subsect. of *Viola* [1].

Rapid advances in the development of DNA techniques have allowed researchers to propose a natural, genealogy-based classification of organisms. The use of molecular data may accelerate the discovery of species. It is recommended to use molecular tools for plant taxonomy, but integrative taxonomy is also important, based on evidence from multiple sources [64,65].

### 4.1. ISSR Markers and Rad-Seq Subsumed V. pubifolia into Genetic Variation of V. palustris

The genetic diversity of *V. pubifolia* results from its mixed reproductive system (cross-pollination via CH flowers, selfing via CL flowers, vegetative propagation by stolons) [9]. The low genetic diversity, confirmed in this study by ISSR markers, is a consequence of the predominance of vegetative propagation and selfing, in agreement with suggestions about the factors influencing the loss of intraspecific genetic diversity [66,67,68,69]. A comparison of the two groups showed that the gene diversity of *V. palustris* was only slightly higher than that of *V. pubifolia* (Table 2). NeighborNet, as well as structure Bayesian analysis, clearly indicated that all *V. pubifolia* samples grouped together with *V. palustris* (Figure 3 and Figure 4).

The previous hypothesis of the origin of *V. palustris* subsp. *pubifolia* via introgression [9] was not confirmed by RAD-Seq, showing the genetic similarity of this taxon to *V. palustris*. Theoretically, backcrosses of F_1_ hybrids (*V. epipsila* × *V. palustris*) to one or both parental species may occur in nature, as the hybrid produces a small percentage of viable pollen [9] (Figure S2).

The deeper insight into genetic population structures offered by RAD-Seq showed higher genetic diversity and heterozygosity in the group containing *V. pubifolia* and *V. palustris* than in the European *V. epipsila* (Table 3 and Table 4), a rare and endangered species in Central Europe and Fennoscandia [10,29,32,33,34,70]. This seems to confirm the hypothesis that the survival of a species in changing environmental conditions potentially declines if genetic diversity and heterozygosity are low [71,72].

The number of common haplotypes of *V. pubifolia* with *V. epipsila* indicates that individuals named *V. pubifolia* should be recognized as *V. palustris*. The origin of an octoploid *V. palustris* via hybridization of the two vicariant parental species—European *V. epipsila* and eastern North American *V. minuscula* (=*V. pallens*)—followed by genome multiplication is connected with climate cooling and glaciations (last 5 Ma). This was possibly able to occupy a new empty niche available after the glacier had receded [1,18]. As multiple origins of allopolyploids (polytopy) are a common phenomenon in nature [73], *V. palustris* may have been created in other regions of Europe, for example, in Lithuania and Germany, based on regional parental species.

### 4.2. What Does the GPI Gene Add to Our View of the Origin of V. pubifolia

*GPI* gene has been used successfully to reconstruct species phylogeny and polyploid evolution in the genus *Viola* L. [1,18,46]. Sequencing two homoeologs of this highly conserved gene from randomly selected samples of *V. pubifolia* subsumed this taxon into the *V. palustris* group (Figure 7). The relationship of *V. pubifolia* with the species of subsect. *Stolonosae* indicated that its origin was the same as *V. palustris* with the involvement of *V. epipsila* subsp. *epipsila* and *V. minuscula* (=*V. pallens*) as parents (Figure 8). *Viola pubifolia,* as well as *V. palustris*, shares homoeologs with both parental species.

### 4.3. Viola palustris—A Highly Morphologically Variable Species

This great morphological variability of *V. palustris* may have resulted from the hybrid origination of this species [*V. epipsila* × *V. minuscula* (*=V. pallens*)], followed by genome duplication [18]. In sexually reproducing *V. palustris* [9], new genotypes generated by meiotic recombination may have features predominantly of one species or the other. A more likely explanation is that parental phenotype A, expressed as a polymorphism in the allopolyploid *V. palustris*, is the result of the knockout or silencing of the B homoeolog. Polyploidization induces genetic and epigenetic processes, including DNA sequence elimination and gene silencing, contributing to the functional diversification or subfunctionalization of duplicated genes, and the genetic and cytological diploidization of allopolyploids. Epigenetic modifications may produce adaptive epimutations and novel phenotypes [74,75,76].

The hairy lower leaf surface and the location of bracts in the middle or even above the middle of the pedicel of *V. pubifolia* individuals observed at CH or CL blooming can lead to erroneous classification to *V. epipsila* or hybrid **V. epipsila* × *V. palustris** [9,10].

It is recommended to describe and classify specimens in the field during the period of CH or CL fruiting. Normally developed, the seed-filled capsules indicate *V. palustris* but not a hybrid, which is sterile. It should also be taken into account that the shape and hairiness of the leaf blade are seasonally variable; there are also differences between leaves developing on stolons and on rhizomes (stolon leaves are more acute and with a more open sinus). Herbarium specimens in the vegetative stage (only leaves and rhizomes) are impossible to be correctly identified in the *V. epipsila*—*V. palustris* group (Elżbieta Kuta and Thomas Marcussen, personal observations during a critical revision of herbarium specimens from Europe).

This study showed the usefulness of molecular markers in species delimitation of the European peatland *Viola* as important for biodiversity and nature conservation. The increased number of interspecific hybrids (*V. epipsila* × *V. palustris*), with a simultaneous reduction in the number of *V. epipsila* and/or *V. palustris* individuals or populations ([10,30] and references therein), can be considered as indicators of anthropogenic changes in the peatlands and their continued degradation.

## 5. Conclusions

(1) Morphological characters of *V. pubifolia* fall well within the range of variability of *V. palustris*.
(2) Genetically, *V. pubifolia* is *V. palustris*, based on ISSR, *GPI*, and RAD-Seq.
(3) The low genetic diversity and heterozygosity of selected Central and Northern European populations of *V. epipsila* confirmed by RAD-Seq might explain its low tolerance to changing environments and the risk of extinction.
(4) The declining number of *V. epipsila* populations and the disappearance of its natural habitats suggest that in some areas it might require effective conservation strategies.

## Figures and Tables

**Table 1:** PCR and sequencing primers used in *GPI* analysis.

PCR Primers (5’-3’) *	Annealing Temperature °C	Sequencing Primers **	Sequenced Gene Region	Homoeolog
Step 1				
Gpi C12Fpcr (TCCAATATGGTTTCTCCATG)/ and Gpi C16Rpcr (AAGTGGTAGACCATCAATAGAA)/	49	Gpi C13Rseq (GCATACACATGCACTTATACC); Gpi cham15R (TAAGATGGCCTGTGAGCAC)	exon12–exon16	-
Gpi M12F (CTCTCCAATATGGTTTCTCCATT)/ and Gpi melvio16R (GAAGTGGTAGACCATCAATAGAT)	58	Gpi M12F; Gpi melvio16R		
Gpi C13Fpcr (CGACTTTAGGTAGATTAAAGTG) and Gpi cham17R (CAACTTCWTGAATCTAAATCTTG)	49	Gpi melvio13R (TTAAAAAACCATAAAGTGTGCATTCC); Gpi melvio15R (TAAGATGGCCTGTGAGCAT)		
Gpi melvio17R (AACTTMTKGAATCTAAAAYCCTC) and Gpi melvio13F (GTCGTGTGGAATTTGCAGG)	49	Gpi melvio17R; Gpi melvio13F	exon13–exon18	-
Step 2				
Gpi C12Fpcr and Gpi C16Rpcr	62.5–49	Cepi_0077F_T (TTCTGAAATTCAT); Cpal_0077F_C (TTCTGAAATTCAC); Cepi_1351R_A (AGAAAAGGAAGGAA); Cpal_1351R_G (AGAAAAGGAAGGCG)	exon12–exon16	CHAM
Gpi M12F and Gpi melvio16R	62.5–49	Mepi_0121F_C (TCATGAGACTAAGC); Mpal_0121F_G (TCATGAGAATAAGG); Mepi_1313R_T (CCTGTTGAATATGT); Mpal_1313R_C (CCTGTTGAATATGC)		MELVIO
Gpi C13Fpcr and Gpi cham17R	62.5–49	Cepi_1079F_G (ATCTTGTCTTATTG); Cpal_1079F_T (ATCTCGTCTTATTT); Cepi_2065R_C (AAATCGGAGGGAAC); Cpal_2065R_T (AAATCGGAGGGAAT)	exon13–exon17	CHAM
Gpi melvio17R and Gpi melvio13F	62.5–49	Mepi_1083F_G (TGTCGTATTGTTTG); Mpal_1083F_T (TGTCGTATTGTTTT); Mepi_2030R_G (AGATGCGTTAACCG); Mpal_2030R_A (AGATACGTTAACCA)		MELVIO

* PCR primers from steps 1 and 2 according to Marcussen et al. [18]. ** Sequencing primers from step 1 according to Marcussen et al. [18]; sequencing primers from step 2 designed according to Scheen et al. [47] protocol.

**Table 2:** Parameters of genetic diversity based on Dice coefficient from ISSR data: A—*V. pubifolia* (20 samples) and *V. palustris* (15 samples); B—*V. pubifolia* (62 samples); N—number of specimens used in genetic analyses; N_poly_—number of polymorphic markers; %_poly_—proportion of polymorphic markers, *H_j_*—Nei’s [62] gene diversity; *H_T_*—total gene diversity; *H_S_*—mean gene diversity within populations; *G_ST_*—Nei’s [62] gene diversity between populations.

	Pop	N	N_poly_	%_poly_	*H_j_*	*H_T_*	*H_S_*	*G_ST_*
**A**	SPN	20	36	17.91	0.05	0.09	0.07	0.21
	PAL *	15	54	26.87	0.09			
**B**	SPN	62	62	30.85	0.06			

* PAL population contains *V. palustris* from Germany, Poland, Lithuania, and Norway. Detailed information in Table S1.

**Table 3:** Marker parameters for all sites of studied *Viola* taxa based on RAD-Seq analysis results. N_priv_—number of private alleles in population; N_Indv_—mean number of individuals per locus in population; N_poly_—number of polymorphic sites; %_poly_—percentage of polymorphic sites; P—mean frequency of the most frequent allele at each locus in population; *H_o_*—mean observed heterozygosity; Obs. Hom.—mean observed homozygosity; *H_e_*—mean expected heterozygosity; Exp. Hom.—mean expected homozygosity; π—mean nucleotide diversity in population; *F_IS_*—mean inbreeding coefficient of an individual relative to the subpopulation.

Pop ID	N_priv_	N_Indv_	N_poly_	%_poly_	P	*H_o_*	Obs. Hom.	*H_e_*	Exp. Hom.	π	*F_IS_*
Vpub	31	12.16	443	0.28	0.75	0.48	0.52	0.28	0.72	0.29	−0.35
Vpal	24	3.00	417	0.27	0.75	0.46	0.54	0.28	0.72	0.33	−0.21
Vepi	14	3.00	40	0.03	0.98	0.02	0.98	0.02	0.98	0.03	0.02
hybrid	8	2.00	581	0.37	0.64	0.72	0.28	0.39	0.61	0.52	−0.29

Vpub—*V. pubifolia*, Vpal—*V. palustris*, Vepi—*V. epipsila*, hybrid—*V. epipsila* × *V. palustris*.

**Table 4:** Pairwise SNP-level *Fst* distance between species and interspecific hybrids based on RAD-Seq analysis results.

	Vpub	Vpal	Vepi	Hybrids
Vpub	-	0.03	0.41	0.16
Vpal	0.03	-	0.52	0.16
Vepi	0.41	0.52	-	0.33

Vpub—*V. pubifolia*, Vpal—*V. palustris*, Vepi—*V. epipsila*, hybrid—*V. epipsila* × *V. palustris*.

**Table 5:** Number of common haplotypes of Viola pubifolia with *V. epipsila* and *V. palustris* based on RAD-Seq analysis results.

*V. pubifolia* Sample Name	No of Common Haplotypes with Selected Taxa or Sample							
	All *V. epipsila* Rs16–18	*V. epipsila* from Poland Rs16	*V. epipsila* from Poland Rs18	*V. epipsila* from Lithuania Rs17	*V. palustris* from Germany Rs14	*V. palustris* from Lithuania Rs15	All *V. palustris* Rs14,15,21	All Hybrids Rs19,20
Rs1	107	122	124	127	532	605	478	302
Rs2	110	126	126	130	536	608	481	307
Rs3	90	104	105	107	485	543	434	266
Rs4	102	118	118	122	520	587	471	288
Rs5	101	116	118	121	526	591	472	291
Rs6	87	100	105	107	502	560	454	277
Rs7	99	115	115	118	495	573	448	273
Rs8	92	105	110	113	511	572	459	287
Rs9	96	107	115	115	516	574	465	305
Rs10	108	121	125	127	502	569	450	277
Rs11	106	122	122	125	496	573	450	279
Rs12	99	113	115	118	515	572	463	279
Rs24	93	107	111	110	552	581	472	281
**Mean**	99.23	113.54	116.08	118.46	514.46	577.54	461.31	285.54

Rs1–Rs24—detailed information in Table S1.

## Data Availability

The sequences of two homoeologs of *GPI* (CHAM—*GPI-C*, MELVIO—*GPI-M*) of *V. palustris* were deposited in GenBank with accession numbers OM681348–OM681351. Raw RAD-seq reads were deposited in Sequence Read Archive and are available under NCBI BioProject with accession number PRJNA807904.

## Supplementary Materials

The following supporting information can be downloaded at: https://www.mdpi.com/article/10.3390/biology12030362/s1, Table S1: Plant material of *V. pubifolia*, *V. epipsila*, *V. palustris* and hybrids **V. epipsila* × *V. palustris** for molecular analyses: genome size value; ISSR markers; *GPI* gene; RAD-Seq; Table S2: Comparison of genome size of selected specimens of *V. pubifolia*, *V. palustris*, hybrids **V. epipsila* × *V. palustris**, and *V. epipsila*; Table S3: Comparison of features of *V. pubifolia*, *V. epipsila*, *V. palustris*, interspecific hybrid (**V. epipsila* × *V. palustris**) based on selected European floras and publications; Table S4: Sequences of used ISSR primers and their annealing temperatures; Figure S1: Values of ΔK for each run with assumed K steps from 1 to 5; Figure S2: Hypothetical model of *V. pubifolia* origin.
