# Supplementary material for: Genetic Variation versus Morphological Variability in European Peatland Violets (Viola epipsila—V. palustris Group)
Source: Biology (Basel). 2023 Feb 24;12(3):362. doi: 10.3390/biology12030362 (PMC10045548; doi:10.3390/biology12030362)
Supplement: Supplementary file 1 [file biology-12-00362-s001.zip › Figure S2.pdf]

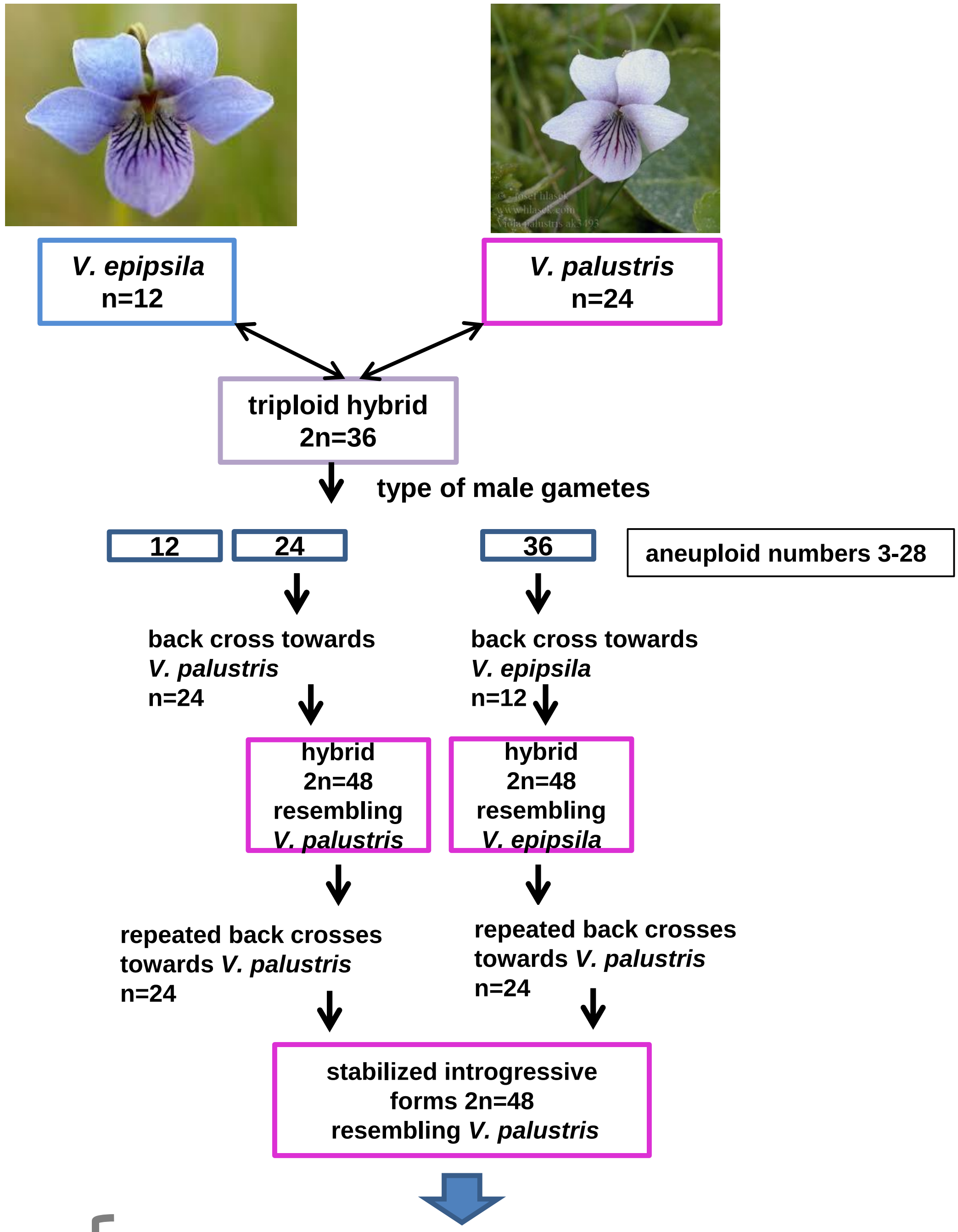

Basionym: *Viola palustris* subsp. *pubifolia* Kuta
Fragm. Flor. Geobot. 35: 21, 1991

***Viola pubifolia* (Kuta) G.H. Loos,**
comb. et stat. nov.,
Jahrb. Bochum. Bot. Vereins 1: 130, 2010

Based on Kuta 1989: Biosystematic studies on the genus *Viola* L., section Plagiostigma Godr. II. Embryological analysis of *V. epipsila* Ledeb., *V. palustris* L. and their hybrids from Poland at: https://www.researchgate.net/publication/268218935
