# Supplementary material for: Genetic Variation versus Morphological Variability in European Peatland Violets (Viola epipsila—V. palustris Group)
Source: Biology (Basel). 2023 Feb 24;12(3):362. doi: 10.3390/biology12030362 (PMC10045548; doi:10.3390/biology12030362)
Supplement: Supplementary file 1 [file biology-12-00362-s001.zip › Table S1.pdf]

Table S1. Plant material of *V. pubifolia*, *V. epipsila*, *V. palustris* and hybrids *V. epipsila* × *V. palustris* for molecular analyses: genome size value; ISSR markers; *GPI* genes; RAD-Seq.

Taxon	Designation of samples	Taxon identification	Localities	Geographical coordinates	Collector**	Used for particular analysis (+)			
						**2C-DNA**	**ISSR**	***GPI***	**RAD-seq**
Viola pubifolia	Rs1-Rs12	genome size, observations in the field by J&PŻ based on previously published morphological features (Kuta 1991 [10])	Słowiński National Park, Poland NW	54.74933, 17.46275	JŻ, PŻ				+
	SPN1-21, 23-57, 59-61, 63-64, SPN67					+	+	+	
	SPN68-SPN86					+			
	Rs24 (=N_B!6)	observations in the field by RH based on morphological features, samples also used in Żabicka et al. 2020 [30]	Floß, Germany SE	49.712330, 12.238800	RH				+
	N_B!2*, N_B!3*, N_B!5*	observations in the field by RH based on morphological features, samples also used in Żabicka et al. 2020 [30]	Floß, Germany SE	49.712330, 12.238800	RH		+		
Viola palustris	BAL_7*, BAL_9*, BAL_10*	based on Żabicka et al. 2020 [30]	Balinka, Poland NE	53.806423, 23.172279	EK		+		
	Rs14 (=N_B3)		"Dormitzer Forst" Kalchreuth, Germany SE	49.565470, 11.109230	RH				+
	N_E1, N_E2	observations in the field by FS based on morphological features	Grillenburg, Germany E	50.940814, 13.497555	FS		+		
	N_E3		Oberlausitz, Germany E	51.202770, 14.849602			+		
	Rs15 (=L_18*), L_19*, L_20*	based on Żabicka et al. 2020 [30]	Pravalas Botanical Reserve, Lithuania E	54.958060, 25.676570	ZG		+		+
	NO1, NO2, NO3	genome size, observations in the field by AR based on morphological features	Barmøya, Norway SW	62.005556, 5.261216	AR	+	+		
	Rs21	genome size, observations in the field by ZC, PSZ, JŻ, PŻ, based on morphological features	Połajewo near Poznań, Poland W	52.733254, 16.735284	ZC, PSZ, JŻ, PŻ	+			+
Viola epipsila	Rs16 (=SZ_4*), SZ_6*, SZ_7*	based on Żabicka et al. 2020 [30]	Szczupliny, Poland NE	53.374917, 20.001750	LM		+		+
	Rs17 (=L_1)		Pravalas Botanical Reserve, Lithuania E	54.958060, 25.676570	ZG				+
	Rs18 (=BPN1)		Białowieża National Park, Poland NE	52.778390, 23.856920	JŻ, LM, AK				+
Hybrids F_1	Rs19 (=BPN2)		Białowieża National Park, Poland NE	52.778390, 23.856920	JŻ, LM, AK				+
	Rs20 (=OST_2), OST_1*, OST_3*, OST_5*		Ostrów Tarczyński Nature Reserve, Poland NE	53.339667, 19.889250	LM		+		+
**Number of sample used: 121**									

*In this study, isolated DNA samples were used from Żabicka et al. 2020 [30]; in parentheses - samples designed as in Żabicka et al. 2020 [30]

Rs – DNA samples used for RAD-seq

**EK – Elżbieta Kuta, RH – Rudolf Höcker, JŻ – Justyna Żabicka, PŻ – Piotr Żabicki, ZC - Zbigniew Celka, PSZ - Piotr Szkudlarz, LM – Leszek Mackiewicz, AK –Andrzej Keczyński, ZG – Zigmantas Gudžinskas, FS – Friedrich Sander, AR – Adam Rostański
