# Supplementary material for: Genetic Variation versus Morphological Variability in European Peatland Violets (Viola epipsila—V. palustris Group)
Source: Biology (Basel). 2023 Feb 24;12(3):362. doi: 10.3390/biology12030362 (PMC10045548; doi:10.3390/biology12030362)
Supplement: Supplementary file 1 [file biology-12-00362-s001.zip › Table S2.pdf]

Table S2.

Comparison of genome size of selected specimens of *V. pubifolia, V. palustris*, hybrids *V. epipsila* × *V.palustris,* and *V. epipsila*.

Taxon	Mean 2C DNA (pg) (±SD)	1C/Cx (pg)
V. pubifolia (N=39)	4.14 (±0.14)[a]	2.07/0.52
V. epipsila (N= 3)	2.52* (±0.03)[b]	1.26/0.63
V. palustris (N= 4)	4.26* (±0.02)[a]	2.13/0.53
Hybrid (N=15) (*V. epipsila* × *V. palustris*)	3.42* (±0.06)[c]	1.71/0.57

Average values followed by the same letter do not differ significantly at $P \leq 0.05$ in Kruskal-Wallis test

*Data taken from Żabicka et al. 2020 [30].
