# Supplementary material for: Genetic Variation versus Morphological Variability in European Peatland Violets (Viola epipsila—V. palustris Group)
Source: Biology (Basel). 2023 Feb 24;12(3):362. doi: 10.3390/biology12030362 (PMC10045548; doi:10.3390/biology12030362)
Supplement: Supplementary file 1 [file biology-12-00362-s001.zip › Table S3.pdf]

Table S3. Comparison of features of *V. pubifolia*, *V. epipsila*, *V. palustris*, interspecific hybrid (*V. epipsila* × *V. palustris*) based on selected European floras and publications.

Feature		*V. epipsila*	*V. palustris*	Hybrid F_1 (*V. epipsila* × *V. palustrs*)	*V. pubifolia*
**leaf**					
- length/width ratio		0.51–1.50 (f, h)	**0.54–1.17** (f, h)	0.62–1.36 (f, h)	**0.58–0.87** (f)
- pubescence of the lower surface	hairy	+ (a, b, c, f, g,h)	+ (b, g)	+ (f, h)	+ (f, i)
	glabrous		+ (a, b, f, g, h, c)	+ (f, h)	
- shape of apex	acute	+ (b, f, h)	+ (a, b, h)	+ (h)	
	subacute	+ (b, g, h)	+ (b, h)	+ (f, h)	
	rounded or obtuse	**+ (b, g, h)**	+ (a, b, c, f, g, h)	+ (f, h)	+ (f, i)
- type of margin	serrate	+ (f)			
	crenate	+ (b, g, h)	+ (a, b, f, g, h)	+ (f, h)	+ (f, i)
**flower**					
- length of CH sepals with calycine appendages (mm)		4.5–8.0 (f, g, h)	**3.5–7.0** (f, g, h)	4.0–7.5 (f, h)	**3.5–5.0** (f)
- length of CH spur (mm)		2.5–3.5 (g, f)	**0.5–1.5** (f, g)	1.0-3.0 (f)	**1.0–1.5** (f)
- length of CH lower petal with spur (mm)		12–23 (f, g, h)	**8–14** (f, g, h)	10-16 (f, h)	**9–12** (f)
- location of the bracts on the pedicel	ratio of distance of bracts from rhizome to length of pedicel	0.50–0.93 (f, h)	**0.20–0.60** (f, h)	0.32–0.70 (f, h)	**0.31–0.66** (f)
	in the lower half of the pedicel		+ (b, c, g)		
	in the middle of the pedicel or slightly above		+ (b, c)		+ (i)
	in the upper half of the pedicel	+ (a, b, c, g)			+ (i)
- pubescence of CH lateral petals	hairy	+ (b, f, h)	+ (f, g, h)	+ (f, h)	+ (f, i)
	glabrous	+ (b, f, g, h)	+ (g)		
- pubescence of pedicel	hairy (scattered hairs)	+ (g, i)	+ (g, i)	+ (i)	+ (i)
	glabrous	+ (g, i)	+ (g, i)	+ (i)	+ (i)
- pubescence of petiole	hairy (scattered hairs)	+ (g, i)	+ (g, i)	+ (i)	+ (i)
	glabrous	+ (g, i)	+ (c, g, i)	+ (i)	+ (i)
**karyology and embryology**					
- chromosome number (2*n*) /ploidy level (*x*)		24/4*x* (d, g)	**48/8*x*** (d, g)	36/6*x* (d)	**48/8*x*** (d)
- fruits (capsules) filled with seeds (fully fertile); female line development regular		+ (d, e, h, i)	+ (d, e, h, i)		+ (e, i)
- lack of fruits (fully sterile) female line development disturbed				+ (d, e, h, i)	
- pollen viability (stainbility)	viable (80-100 %); male meiosis regular	+ (e, i)	+ (e, i)		+ (e, i)
	0-30% viable pollen grains, male meiosis disturbed, pollen grains cytologically differentiated			+ (e, i)	

a – Hegi 1925 [23]; b – Zabłocki 1947 [63]; c – Valentine et al. 1968 [26]; d – Kuta 1989a [8]; e – Kuta 1989b [9]; f – Kuta 1991 [10]; g – Karlsson and Marcussen 2010 and references therein [17]; h – Żabicka et al. 2020 [30]; i – Żabicka personal observations in the nature and in cultivation. CH – chasmogamous flowers.
