# Supplementary material for: Genetic Variation versus Morphological Variability in European Peatland Violets (Viola epipsila—V. palustris Group)
Source: Biology (Basel). 2023 Feb 24;12(3):362. doi: 10.3390/biology12030362 (PMC10045548; doi:10.3390/biology12030362)
Supplement: Supplementary file 1 [file biology-12-00362-s001.zip › Table S4.pdf]

Table S4. Sequences of used ISSR primers and their annealing temperatures.

Primer	Primer sequence (5'-3')	Annealing temperature (°C)
ISSR1	5'-TCTCTCTCTCTCTCTCC-3'	50.2
ISSR2	5'-AGAGAGAGAGAGAGAGT-3'	49.9
ISSR3	5'-GGGTGGGGTGGGGTG-3'	53
ISSR4	5'-ATGATGATGATGATGATG-3'	44
ISSR5	5'-ACACACACACACACACG-3'	48.6
ISSR6	5'-ACACACACACACACACT-3'	42.6
ISSR7	5'-GAGAGAGAGAGAGAGAYG-3'	48
ISSR8	5'-ACACACACACACACACYC-3'	50.3
ISSR9	5'-GACAGACAGACAGACA-3'	44
ISSR10	5'-ACTGACTGACTGACTG-3'	44
