# Supplementary figures and images for: Genetic Variation versus Morphological Variability in European Peatland Violets (Viola epipsila—V. palustris Group)
Source: Biology (Basel). 2023 Feb 24;12(3):362. doi: 10.3390/biology12030362 (PMC10045548; doi:10.3390/biology12030362)

$$\text{DeltaK} = \text{mean}(|L''(K)|) / \text{sd}(L(K))$$

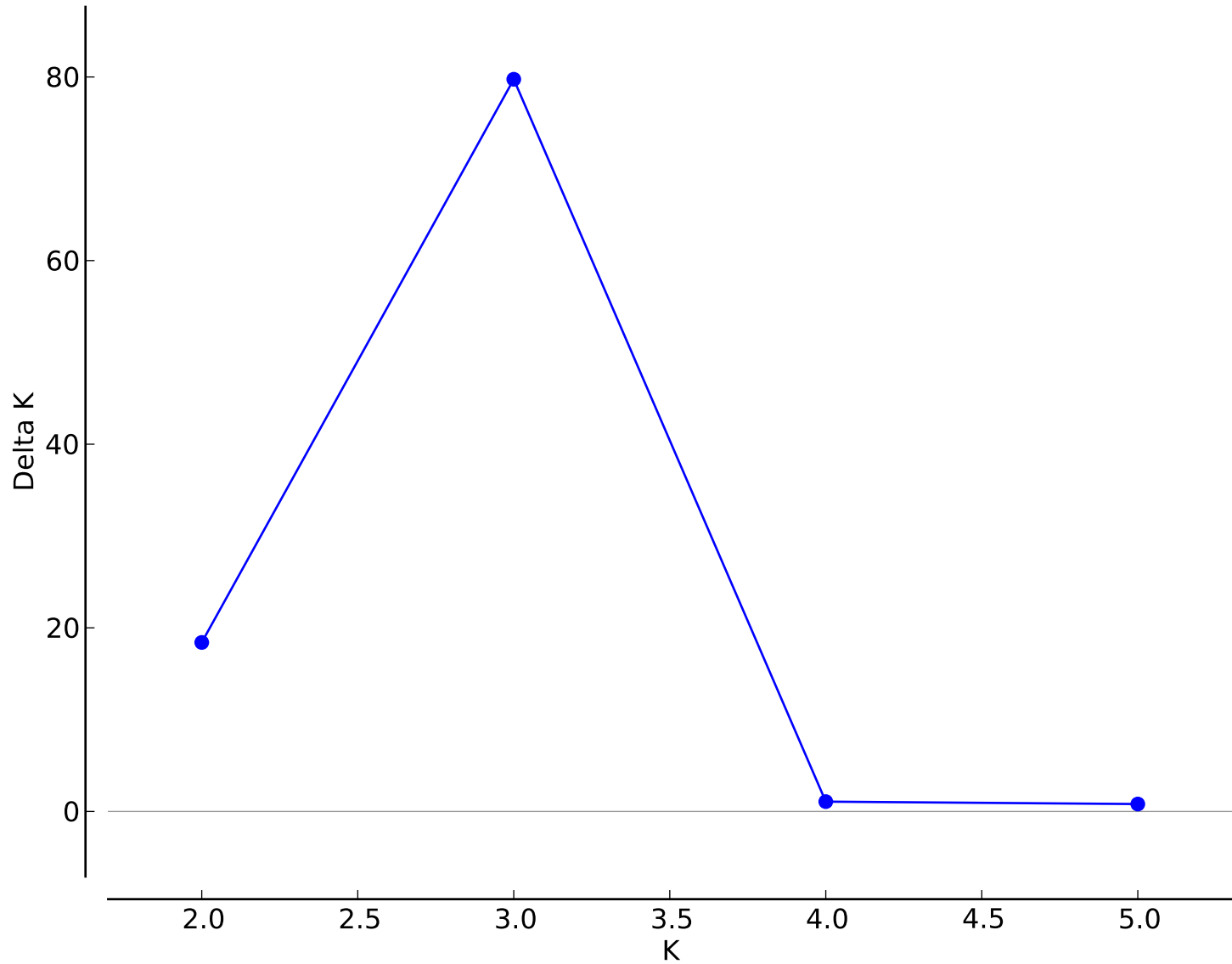

Supplement: Supplementary file 1 [file biology-12-00362-s001.zip › Figure S1.pdf]
